# Modernizing the Methods and Analytics Curricula for Health Science Doctoral Programs

**DOI:** 10.3389/fpubh.2020.00022

**Published:** 2020-02-13

**Authors:** Ivo D. Dinov

**Affiliations:** ^1^Statistics Online Computational Resource, Department of Health Behavior and Biological Sciences, University of Michigan, Ann Arbor, MI, United States; ^2^Neuroscience Graduate Program, University of Michigan, Ann Arbor, MI, United States; ^3^Michigan Institute for Data Science, University of Michigan, Ann Arbor, MI, United States; ^4^Department of Computational Medicine and Bioinformatics, University of Michigan, Ann Arbor, MI, United States

**Keywords:** doctoral training, health science, graduate curricula, methods, analytics, data science, quantitative education

## Abstract

This perspective provides a rationale for redesigning and a framework for expanding the graduate health science analytics and biomedical doctoral program curricula. It responds to digital revolution pressures, ubiquitous proliferation of big biomedical data, substantial recent advances in scientific technologies, and rapid progress in health analytics. Specifically, the paper presents a set of common prerequisites, a proposal for core computational and data analytic curriculum, and a list of expected outcome competencies for graduates of doctoral health science and biomedical programs. The manuscript emphasizes the necessity for coordinated efforts of all stakeholders, including trainees, educators, academic institutions, funding agencies, and policy makers. Concrete recommendations are presented of how to ensure graduates with terminal health science analytics and biomedical degrees are trained and able to continuously self-learn, effectively communicate across disciplines, and promote adaptation and change to counteract the relentless pace of automation and the law of diminishing returns.

## Background

Rapid advances in biomedical research and health science discoveries impact all human experiences. Further progress in this extremely interdisciplinary field requires reexamining policies, funding mechanisms, institutional organizations, graduate education, and training curricula, as well as financial incentives and distribution of limited health resources and services. Future graduates of quantitative biomedical and health science analytics graduate programs will play important roles in legislation, population-wide healthcare policies, and the economic, social, and behavioral determinants of human health. Recent computational, data science, and communication breakthroughs present an opportunity to review and enhance the terminal biomedical and health curricula by adopting modern scientific methods, embracing artificial intelligence analytical strategies, and emphasizing reproducible one-science computational protocols.

Graduates of doctoral biomedical and health science programs should be prepared to continuously self-learn, play active roles in research, participate in health policy, and engage in transdisciplinary collaborations. To be successful in these endeavors, a level of prerequisites is required prior to enrollment in the programs, and the expectations of freshly minted scholars should include technical competencies and transdisciplinary skills to ensure their long-term career success.

### Key Points

- None of the health science disciplines (e.g., medicine, nursing, pharmacy, kinesiology, public health, bioinformatics, and genetics) are insular (1–3). Transdisciplinary training and inter-professional education are critical for ethical, collaborative, and reproducible research of complex biomedical and health conditions (4, 5).
- The digital revolution demands substantial quantitative skills, data-literacy, and analytical competence: health science doctoral programs need to be revised and expanded to build basic-science (STEM) expertise, emphasize team-science, rely on holistic understanding of biomedical systems and health challenges, and amplify dexterous abilities to handle, interrogate, and interpret complex multisource information (6–8).
- Exploratory, classification, and predictive Big Data analytics are all pivotally important and complementary to traditional hypothesis-driven confirmatory analyses (9–12).

### Why Is Curriculum Redesign Important?

- Graduates of health science doctoral programs *may not be fully prepared* to participate in, or lead, transdisciplinary translational research projects involving complex information and heterogeneous observations (13–16).
- The range of *quantitative expertise* in computational and data sciences varies substantially within and between programs (17, 18).
- The NIH-wide 2016–2020 Strategic Plan calls for core data science training and the need for “*quantitative and analytical approaches, processes, and systems … to extract knowledge and insights from increasingly large and/orcomplex sets of data*” (https://datascience.nih.gov/sites/default/files/NIH_Strategic_Plan_for_Data_Science_Final_508.pdf).
- In health sciences, having a STEM foundation knowledge, possessing high technical skills, and having the abilities to gather, model, process, and interpret large amounts of heterogeneous, multi-source, and time-varying information is *gold*. The *golden rule* in advanced evidence-driven patient care, effective biomedical research, and transformative health science is simple: whoever has the gold will make the rules, control the direction of health science, and dominate the translational biomedical research impact. This golden rule is more pertinent to teams of investigators, rather than individual scientists, although the latter form the building blocks of all highly effective teams (19, 20).

### Targeted Trainees

The extremely wide range of graduate biomedical, informatics, and health analytics training programs is a direct reflection of the disruptive nature of network-science based discovery, technological advances, and accelerated data-driven innovation (21–23). This manuscript addresses the need for educating and training a very specific cohort of data-savvy quantitative scholars pursuing terminal research-intensive degrees in biomedical and health sciences. Examples of such trainees include students enrolled in doctoral programs in health informatics, biomedical informatics, biostatistics, human genetics, data science, biomathematics, applied statistics, biomedical engineering, pharmacogenomics, and health analytics. This paper does not reflect on the curricular demands, or the quantitative training, of physicians, practicing clinicians, qualitative biosocial scholars, or licensed healthcare providers who are primarily focused on healthcare delivery. At the same time, some of the proposed technical training may be very appropriate for such practitioners as it will allow them to acquire additional skills, promote effective translation of STEM science and advanced analytics into clinical practice, and potentially improve health outcomes, job satisfaction, and patient experiences. Just like quantitative data scientists must possess dexterous artistic skills (8), it's reasonable to assume that exceptional clinicians will have functional quantitative abilities, and productive biomedical scholars would have basic anatomical and health training.

### Big Biomedical and Health Data

#### Characteristics of Big Health Data

Over a decade ago, academic and IBM researchers introduced the qualifying notion of 3Vs of Big Data (volume, velocity, and variety), which later was expanded to 7Vs by adding veracity, variability, value, and visualization (24–26). This earlier framework provided a qualitative formulation expressing challenges related to the emergence and deluge of big biomedical and health data. Our more quantitative approach is formulated by examining dozens of challenging contemporary biomedical case-studies involving complex biomedical and healthcare datasets. There are seven dimensions of Big Biomedical and Health Data–size, format complexity, observation heterogeneity, incompleteness, spatiotemporal variability, multisource components, and multiscale resolution (9, 27). As a proxy of the underlying complex biological, physiological, and medical conditions, such data are important to understand the causes of morbid conditions, model associations between factors, predict risks of treatments, and forecast clinically relevant outcomes. Examples of big biomedical datasets include the UK Biobank (UKBB) (28–30), the Human Connectome Project (HCP) (31, 32), and the Alzheimer's Disease Neuroimaging Initiative (ADNI) (33, 34). UKBB represents a survey of a large population-based cohort including about 500 K individuals assessed at 22 UK medical centers in UK between 2006 and 2010. National Health Service recipients were invited to participate in UKBB and included individuals mostly between 40 and 69 years old (30, 35). HCP includes behavioral data, clinical phenotypes, and unprecedented high-resolution multimodal neuroimaging data for over 1,000 young adults (36). ADNI collected serial data for several thousands of participants including imaging (e.g., sMRI/fMRI, dMRI, PET), biological markers, clinical, genetics, cognitive, and neuropsychological assessment to measure the disease progression from normal aging to mild cognitive impairment (MCI) and early dementia (33). All of these large-scale studies face a number of challenges like balancing the (large) sample sizes with (small) effect sizes, incongruences, heterogeneity, time variability, and confounding effects. Once such datasets are represented as computable objects, data analytical strategies to extract valuable information and build actionable knowledge include model-based prediction vs. model-free inference, multiple comparison problems, and reproducibility (12, 27, 37).

#### Successes and Failures

Innovation is by definition uncertain and risky! The future of biomedical and health science discovery is bright and there are bound to be spectacular failures as well as breathtaking triumphs. Skeptics may point that major challenges of big data-driven transdisciplinary discoveries include communication barriers and the potential for bias inherent to dealing with complex and voluminous information. Others may argue that the quantity of observed data may obfuscate the key scientific questions transforming the traditional hypothesis-based (confirmatory) research based on a priori observations and inquiries into a new paradigm of data-driven inference, empirical knowledge derivation, and the formulation of novel hypotheses. The 2011 Google Flu Trends (GFT) report (38) was an example where GFT prediction problems were identified in 2013 (39) and partially attributed to overfitting. The GFT original report intended to predict future doctor office visits associated with influenza-like illness, which can be compared to the corresponding flu cases reported by the Centers for Disease Control and Prevention (CDC). In February 2013, independent investigators reported significantly higher GFT-predictions relative to the CDC forecast over the same period of time. The GFT model, which was built on 50-million web search terms over 1,152 data points, predicted increased likelihood of web-search terms matching the propensity of the flu. This may be explained by structurally unrelated queries that may have artificially inflated GFT predictions.

There have also been a number of mind-boggling reports representing successful transdisciplinary work that was only possible using enormous amounts of data interrogated by teams of scientists with broad and deep domain expertise using artificial intelligence (40). For instance, BANDIT (Bayesian ANalysis to determine Drug Interaction Targets), represents a novel data-driven paradigm for target identification and drug discovery using multisource big data in a Bayesian machine-learning framework (41). Applying BANDIT on 2,000 different small molecules, scientists identified likely targets and achieved predictive accuracy of 90%, which was an improvement of prior published target identifications. Similarly, a handful of small molecules with no known targets yielded 4,000 new molecule-target predictions. This target identification along with experimental validation using a set of microtubule inhibitors suggested three candidate compounds for cancer cells resistant to state-of-the-art clinical anti-microtubule treatment. Another example of successful biomedical and health application of transdisciplinary strategies to interrogate big data includes machine-learning techniques. To determine the top determinants of a health outcome, researchers discovered interesting combinations of indicators that affect health outcomes (e.g., life expectancy and anxiety disorders) and identified subpopulations representing analogous clinical phenotypes (42). A 2017 Kaggle Data Science Bowl competition offered $1M prize to a team that improved the specificity of automatic lung nodule characterization to improve screening mammography accuracy (43). Fusion algorithms and computational intelligence were used to efficiently process and visualize 40 GB of data in 10-min (44). Patient-centric eHealth ecosystems provide multi-layer architectures integrating connected devices, computing interfaces, and Cloud services to empower handling of complex data and ensure privacy (45).

Outside biomedical and health science, a recent data-driven discovery used partial differential equations to model large-scale time series measurements in Eulerian (spatially fixed sensors) or Lagrangian (dynamically moving sensors) frameworks. The model distinguishes between linear and Korteweg-de Vries equations, and enables discovery of the physical laws and the corresponding parametric spatiotemporal equations where derivations from first-principle derivations may be challenging (46).

## Analytics Health Science Curriculum

Contemporary health science methods and analytics curricula are somewhat out of step with the accelerated scientific and technological advances in the twenty first century. Modernizing the graduate health science education and training will require substantial efforts to blend quantitative computational and data science methods with qualitative approaches, research ethics, and reproducible open science principles. The Data Science and Predictive Analytics (DSPA) course^1^ provides one complete, openly-accessible, and technology-enhanced example of an advanced quantitative graduate course for health sciences.

### Prerequisites

There are expected variations between different biomedical and health science doctoral programs. Student backgrounds, career interests, motivations, expectations, and learning styles present additional levels of anticipated disparities. Although neither necessary nor sufficient, the prerequisites listed in Table 1 serve as a guideline of the foundational knowledge and prior experience that provide the basis for successful completion of a solid quantitative doctoral program in the biomedical and health sciences.

**Table 1:** Examples of prerequisites for strong biomedical and health sciences quantitative doctoral programs.

**Prerequisites**	**Skills**	**Rationale**
Bachelor's degree or equivalent	Prior quantitative methods/analytics training and coding skills	Graduate programs require a basic minimum level of quantitative skills
Quantitative literacy	Undergraduate calculus, linear algebra, numerical methods, introduction to probability, statistics, or data science	These represent fundamentals that are required for most methods and analytics graduate health science courses
Some coding experience	Some academic, training or professional experience in programming or software development	Most practicing bioinformaticians and health analysts need substantial coding experience, e.g., Java, C/C++, HTML5, R, Python, Perl, PHP, SQL/DB
Strong motivation	Substantial current interest for emersion and motivation to pursue long-term quantitative data analytic applications	Dedication for prolonged and sustained immersion into hands-on practice, collaboration, and methodological health research is very important

Potential trainees that have insufficient prior domain expertise, e.g., in college-level mathematics, numerical methods, probabilistic modeling, statistical analysis, or software programming, may need to complete relevant bootcamps or remediation coursework prior to matriculation. A wide range of MOOCs may provide the necessary prerequisites, e.g., Coursera, EdX, Khan Academy, Udacity. Examples of remediation courses provided to satisfy some of the Data Science and Predictive Analytics (DSPA) prerequisites are included in the DSPA self-assessment (pretest).

### Core Curriculum

Indeed, each Institution and each quantitative biomedical or health sciences doctoral program will have their own customized curricula. At the same time, certain types of fundamental topics are expected to be common and share core principles, coverage, and methods. Table 2 illustrates examples of types of computational and data science courses that graduate students^2^ at any of the 12 disciplines part of the Program in Biomedical Sciences (PIBS)^3^ at the University of Michigan choose from. Many of these courses have analogs at other Institutions and attract young scholars interested in data-intense transdisciplinary research, development, and training.

**Table 2:** Exemplary courses at the University of Michigan.

**Courses**	**Descriptions**	**Types**
HS853: Advanced scientific methods for health sciences	Covers a number of modern analytical methods for advanced healthcare research. Specific focus is on reviewing and using innovative modeling, computational, analytic and visualization techniques to address specific driving biomedical and healthcare applications. The course covers the 6 dimensions of Big-Data, statistical cross-validation, model-based, and model-free forecasting	Analytics/applications
HS650: Data science and predictive analytics	Concepts, techniques, tools, and services for managing, harmonizing, aggregating, preprocessing, modeling, analyzing, and interpreting large, multi-source, incomplete, incongruent, and heterogeneous data (Big Data). The focus will be to expose students to common challenges related to handling Big Data and present the enormous opportunities and power associated with our ability to interrogate such complex datasets, extract useful information, derive knowledge, and provide actionable forecasting	Analytics
PIBS503: Research responsibilities and ethics	Covers case-studies on fraud, fabrication, and plagiarism, data storage, ownership, and peer review, animal use and care, human subjects research and IRBs, conflict of interest, research in the global workplace, dual use issues, discussion about ethical practices particular to project/laboratory	Research ethics
BIOINF585: Machine learning for systems biology & clinical informatics	Focuses on machine learning methods and their applications in biomedical sciences. Topics include: (1) data management solutions for Big Data applications, (2) feature extraction and reduction methods, (3) clustering and classification methods, (4) testing and validation of models, (5) applications in systems biology and clinical informatics	Methods and apps
BIOINF527: Introduction to bioinformatics and computational biology	Introduces students to the fundamental theories and practices of Bioinformatics and Computational Biology via a series of integrated lectures and labs. These lectures and labs will focus on the basic knowledge required in this field, methods of high-throughput data generation, accessing public genome-related information and data, and tools for data mining and analysis	Methods and apps
BIOSTAT602: Biostatistical inference	Provides deep understanding of key concepts and analytics of statistical inference. Statistical inference methods are of critical importance for statisticians to properly process data and organize information to quantify uncertainty so to delivery adequate solutions to substantive questions	Methods and analytics
Math 571: Numerical linear algebra	Introduces numerical linear algebra as a core subject in scientific computing. Three types of problems are considered: (1) linear systems (*Ax* = *b*), (2) eigenvalues and eigenvectors (*Ax* = λ*x*), and (3) least squares problems (min_x_ ||*Ax* − *b*||_2_). These problems arise in many scientific applications and we'll study the accuracy, efficiency, and stability of the methods developed for their solution	Methods and analytics
Stats 415: Data mining and statistical learning	Covers the principles of data mining, exploratory analysis and visualization of complex data sets, and predictive modeling. The presentation balances statistical concepts (such as over-fitting data, and interpreting results) and computational issues.	Methods and analytics
Stats 503: Applied multivariate analysis	Presents modern methods of multivariate data analysis and statistical learning, including theoretical foundations, and practical applications. Topics include principal component analysis and other dimension reduction techniques, classification (discriminant analysis, decision trees, nearest neighbor classifiers, logistic regression, support vector machines, ensemble methods), and clustering	Methods and analytics
NERS 590: Methods and practice of scientific computing	Develops the necessary skills to be effective computational scientists and how to produce work that adheres to the scientific method. A broad range of topics are covered including: software engineering best practices, computer architectures, computational performance, common algorithms in engineering, solvers, software libraries for scientific computing, uncertainty quantification, and validation	Methods
EECS 584: Advanced database management systems	Advanced topics and research issues in database management systems. Distributed databases, advanced query optimization, query processing, transaction processing, data models, and architectures. Data management for emerging application areas, including bioinformatics, the internet, OLAP, and data mining. A substantial course project allows in-depth exploration of topics of interest	Methods and analytics
EECS 545: Machine learning	Introduces computer algorithms that can learn from data or past experience to predict well on the new unseen data. In the past few decades, machine learning has become a powerful tool in artificial intelligence and data mining, and it has made major impacts in many real-world applications. This course gives a graduate-level introduction of machine learning and provide foundations of machine learning, mathematical derivation and implementation of the algorithms, and their applications	Methods and analytics
EECS 453: Applied data analysis	Theory and application of matrix algorithms to signal processing, data analysis and machine learning. Theoretical topics include subspaces, eigenvalue and singular value decomposition, projection theorem, constrained, regularized, and unconstrained least squares techniques and iterative algorithms. Applications include image deblurring, ranking of webpages, image segmentation and compression, social networks, circuit analysis, recommender systems, handwritten digit recognition	Methods and analytics

At the most basic level, graduates should receive analytical training in three complementary domains—mathematics, statistics, and engineering. The *mathematical foundations* should emphasize basic understanding of multi-variable calculus, complex variables and functions, linear algebra, matrix computing, differential equations, numerical methods, and optimization. Statistics knowledge should stress practical experience with at least a couple of different statistical computing software packages, understanding of probability theory, distribution functions, and Bayesian inference, as well as parametric and non-parametric statistical tests. Finally, it is important to enhance the graduates' engineering abilities, develop working knowledge of some compiled and interpreted programming languages, data ingestion, management, and visualization.

In addition to quantitative analytical training, all program graduates should be exposed to qualitative and common-sense human-intelligence training including data quality challenges, model interpretability, research ethics, privacy and security, health policy and regulatory guidelines, and implementation research.

- Data quality challenges are always present in big biomedical and health studies, this includes understanding the importance of tracking provenance and assessing data quality, “fitness for use,” completeness, and complexity (47–49).
- Model interpretability and transparency is important to be understood, disclosed, and properly interpreted to contextualize the performance, bias, implementation approach, reported findings, potential limitations, and possible unintended consequences (50).
- Research ethics blends the individual scholar values, e.g., honesty and personal integrity, and treatment of other individuals involved in the research, e.g., informed consent, confidentiality, anonymity, and courtesy (51).
- Information security, and privacy protection training are absolutely necessary and will play a vital role throughout all professional activities of graduates (52).
- The landscape health policies are constantly created and updated to drive healthcare research and influence health achievements. Legislative and regulatory guidelines also impact biomedical and health research (53). These are intendend to standardize and control types of scholarly nad organizational behavior, monitor, and enforce policies and licensing, and accreditation.
- Implementation research amalgamates scienctific research and healthcare practice. It is focused on the creation knowledge that can be applied to improve health policies, clinical programs, medical practice, and the borader public health (54).

Due to substantial heterogeneities in institutional course offerings, depth and breadth of program coverage, and variations in individual backgrounds, learning-styles, and scholarly interests, “one-curriculum-plan-does-not-fit-all.” It's difficult to prescribe one unique curriculum that includes a specific number of courses to complete, a concrete course-series ordering, and a single completion timeframe. In principle, each Health Science doctoral program will comprise a set of core courses, required for all trainees, a complementary set of specialization and elective courses, and alternative practical experiences (e.g., mentored lab rotations, internships, apprentice shadowing, hands-on capstone projects, etc.).

Table 3 outlines some hypothetical curriculum plans that may be customized and adopted in various quantitative graduate health science and analytical programs. The longitudinal flow (columns) and thematic variability (rows) are neither complete, not exhaustive, or mandatory.

**Table 3:** Examples of hypothetical broad-scope 5-year program plans by specialization.

**Broad thematic specializations**	**Annual progression (years)**					
		**Yr1**	**Yr2**	**Yr3**	**Yr4**	**Yr5**
Bioinformatics	Discipline-specificcourses. Common curricula, e.g., rotations, scientific rigor, reproducibility, and ethics	Data science and predictive analytics	Advanced ML/AI	Inter-professional education, Trans-disciplinary collaborations Add-on certificates Electives, specialized courses	Domain-specific AI/ML applications Computational methods, protocol development, and Cloud computing	Data-driven dissertation-topic specific research
Professional schools (e.g., medicine, nursing, pharmacy, kinesiology)		Computing, statistics, math modeling	Data science and health analytics			
Public health, biostatistics		AI/ML techniques	Clinical decision support systems			
Biomathematics		Computational biology, bioinformatics	High-throughput precision health			
Neuroscience		Computational neuroscience, neuroimaging, brain mapping	Data science and predictive analytics			

### Expected Competencies

In addition to their core area of specialization, graduating doctoral students should be expected to have moderate modeling, computational, and analytic competency in at least two of each of the three competency areas listed in Table 4.

**Table 4:** Expected program graduate's competencies.

**Areas**	**Competency**	**Expectation**	**Notes**
Algorithms and applications	Tools	Working knowledge of basic software tools (command-line, GUI based, or web-services)	Familiarity with statistical programming languages, e.g., R or SciKit/Python, and database querying languages, e.g., SQL or NoSQL
	Algorithms	Knowledge of core principles of scientific computing, applications programming, numerical methods, API's, algorithm complexity, and data structures	Best practices for scientific and application programming, efficient implementation of matrix linear algebra and graphics, elementary notions of computational complexity, user-friendly interfaces, string matching
	Application domain	Data analysis experience from at least one application area, either through coursework, internship, research project, etc.	Applied domain examples include: computational social sciences, health sciences, business and marketing, learning sciences, transportation sciences, engineering, and physical sciences
Data management	Data validation and visualization	Curation, Exploratory Data Analysis (EDA) and visualization	Data provenance, validation, visualization via histograms, Q-Q plots, scatterplots (ggplot, Dashboard, D3.js)
	Data wrangling	Skills for data normalization, data cleaning, data aggregation, and data harmonization and registration. Experience with R notebook or Jupyter notebook	Data imperfections include missing values, inconsistent string formatting (“2016-01-01” vs. “01/01/2016,” PC/Mac/Linux time vs. timestamps, structured vs. unstructured data, ASCII vs. binary format, compression, etc.
	Data infrastructure	Handling databases, web-services, Hadoop, multi-source data	Data structures, SOAP protocols, ontologies, XML, JSON, streaming
Analysis methods	Statistical inference	Basic understanding of bias and variance, principles of (non)parametric statistical inference, and (linear) modeling	Biological variability vs. technological noise, parametric (likelihood) vs. non-parametric (rank order statistics) procedures, point vs. interval estimation, hypothesis testing, regression
	Study design and diagnostics	Design of experiments, power calculations and sample sizing, strength of evidence, *p*-values, False Discovery Rates	Multistage testing, variance normalizing transforms, histogram equalization, goodness-of-fit tests, model overfitting, model reduction
	Machine learning	Dimensionality reduction, k-nearest neighbors, random forests, AdaBoost, kernelization, SVM, ensemble methods, CNN	Empirical risk minimization. Supervised, semi-supervised, and unsupervised learning. Transfer learning, active learning, reinforcement learning, multiview learning, instance learning

One important point to emphasize is that in addition to the proposed quantitative outcomes of any graduate biomedical and health training program, trainees should be expected to acquire a number of complementary qualitative skills. Such abilities include transitional science expertise, behavior change adoptability, and aptitude for identification of significant findings for clinical implementation. The focus of this specific manuscript is on the quantitative part of the training, i.e., the methods and analytics curricula for health science doctoral programs; however, soft skills, human intelligence, and artistic abilities are also important (8).

## Conclusions

The role of continuous self-learning is paramount in the future on-demand economy, where rapid developments and technological advances quickly render static technical skills obsolete. One of the best lessons biomedical and health science doctoral program graduates should learn is the value of sustained lifelong commitment to learning, retooling, knowledge refreshing, and dynamic skill building. This is neither easy, quick, nor necessarily intuitive; however, it is absolutely essential for a perpetually successful career. The main factors driving the need for sustained self-learning include the relentless pace of automation (55), world-wide competition and the rise of the rest (56), the growth of network-based team science (57), the unrelenting anticipation of progress and increase of human well-being over time (58, 59), and the law of diminishing returns (60). The latter asserts that as equal efforts, resources or infrastructure are provided to support an R&D activity, the resulting output from these endeavors will initially increase monotonically with the input up to a certain point, after which, adding additional resources will result in steadily and disproportional decrease where the incremental additive outcome will tend to zero (61).

In addition to the technical, methodological, and analytical skills, there are other qualitative abilities skills that all premier graduate health and biomedical programs should emphasize. As health sciences are both deep and broad in scope, consideration needs to be made to improve inter-professional training and interdisciplinary collaborations (62, 63). Ability to communicate across disciplines is vital to establish, grow and sustain team science, crowdsourcing accomplishments, and citizen scholars, which recently demonstrated forward advances (57). For instance, the Galaxy Zoo project had over 250,000 contributors (Zooites) that completed about 200 million classifications of distance images from the Sloan Digital Sky Survey (SDSS), and over 200,000 users contributed to the Foldit project aiming to quickly enhance our understanding of protein folding via a computer game platform. Active and constructive participation in transdisciplinary teams will require well-rounded background with sufficient depth in specific scientific area and ability to broadly communicate with other experts.

It is undeniable that we need to reorganize the graduate health education and biomedical research training to keep up with the exponential increase of information, the broad knowledge field interactions, and the expeditious technological advances. The broader academic community needs to respond to this digital revolution challenge by balancing the need to preserve basic science rigor at the same time strongly emphasizing transdisciplinary network team-science. As no two programs are the same and there will be enormous progress ahead, there is a need for constant community-based revisions and expansions of the advanced quantitative health science analytics curriculum. All such programs will require environment-specific implementations and the need for contributions from all stakeholders (students, instructors, funding agencies, institutional leaders, and program directors).

It is hard to predict what specific recommendations may guarantee long-term success because the two key components of innovation are *uncertainty* and *risk*. However, aversion to either of these would virtually guarantee colossal failures. Coordinated efforts by policy makers, funding organizations, academic institutions, graduate biomedical, and health science curriculum committees, course instructors, and trainees will be vital to meet the demand for effective, fair, and consistent progress in improving human well-being and enhancing human experiences. Foundations and scholarly work funding agencies should diversify the pool of peer reviewers, embrace risky and unconventional approaches, reduce their multilevel bureaucracy (e.g., on-demand dynamic program staff selection and proposal formatting barriers), and acknowledge serendipity in scientific discovery (64).

There is an urgent need for strong commitment from all stakeholders to increase the availability of data, access to compute resources, open-science principles, and their embedding directly into all graduate program curricula. Improving the efficiencies of data acquisition, utilization of rich and diverse computational protocols, and research ethics training should augment the core program coursework. These burdens fall primarily on non-student stakeholders, e.g., instructors, advisors, curriculum committees, institutional administration, state and federal regulators, and policymakers. Careful planning and thoughtful implementation would be critical to avoid extreme and unreasonable policies, limit the unexpected consequences, and reduce unconstructive overregulation.

It is important to point out that curriculum design and its effective implementation are two separate aspects of equal importance. Deficiencies in either of these will strongly impact the final program and potentially lead to very different outcomes. The success of any graduate academic program redesign depends on many different factors including (1) the specific curriculum design plan, (2) sustained faculty engagement, (3) long-term financial support, (4) strong institutional backing, (5) appropriate trainee prescreening and selection, and (6) organizational infrastructure. It is impossible to make specific recommendations on the required levels of commitment for each of these vital components to “guarantee” successful launch and sustained programmatic triumph. Neither financial backing, infrastructure, expertise, or organization environment is by itself necessary or sufficient for establishing a successful program. The exact blend of these factors that leads to an exceptional quantitative graduate health science methods-and-analytics program will vary. In some institutions, funding may be more important than infrastructure. In others, existence of appropriate computational services or reliable lab equipment may be more influential than candidate prescreening. However, strengths in more than one of these six factors would certainly increase the likelihood of a successful and lasting curricula implementation. Finally, the role of the program teaching, research, and practice faculty, along with their continuing (re)training, strategic recruitment, cultivation, and retention cannot be overestimated.

Federal, state and local public officials should enact egalitarian policies that stimulate research, innovation, development, and productization without compromising individual privacy, research ethics, or sensitive information. The academic institutions that embrace diverse financial endowments, without compromising impartiality, and implement strategies to democratize transdisciplinary collaborations will likely reap substantial benefits and chart the course forward. Individual instructors should adapt open-science principles in their courses, collaborate and share with others their learning modules, source materials, and champion direct connections to other courses, disciplines, techniques, or learning resources. Last but certainly not least, trainees represent the focal point and the future of the effort to enhance the capability and capacity of the biomedical and health workforce. Graduating students should realize that the era of the 9-to-5, long-term job-security, repetitive occupations, and stagnant knowledge career paths ended as the twentieth-century came to a close. Top graduate biomedical and health educational institutions will provide the fundamentals and train scholars how to self-learn, utilize Cloud-knowledge resources, and expand their know-how. The rest is up to individual researchers, their close scholarly networks, and the administrative staff that manages research, development, and translation activities. The lead article in a recent issue of the Economist, “Doctor You: How Data will transform Health Care” (65), predicts an upcoming health care digital revolution that will empower patients, improve diagnosis, lower costs, and introduce apps as alternatives to conventional drugs. However, this sea change is only possible when networks of well-trained researchers jointly design, implement, support, and continuously expand advanced clinical decision support systems.

The stakes of failing to restructure doctoral biomedical and health science education are high for two reasons. The first corresponds to failure of raising a cadre of computationally skilled and data-literate researchers to support the innovation backbone of future healthcare and biomedical discoveries. Second, there will be a very substantial loss-of-opportunity cost associated with lack of appreciation for the urgent need to change quantitative graduate biomedical education. In 1746, in his “Golden Rules” for “Young Tradesman,” Benjamin Franklin wrote that “time is money” (66), referring to idleness as a direct loss. The analog for this eighteenth century work-lethargy loss of revenue, translates in the twenty first century as a societal deficit of equitable, effective, and progressive human health experiences, due to vegetative investment of resources or lackadaisical education vision. The golden rule for the future young biomedical and health science scholars may be “time is life.”

## Author Contributions

The author confirms being the sole contributor of this work and has approved it for publication.

### Conflict of Interest

The author declares that the research was conducted in the absence of any commercial or financial relationships that could be construed as a potential conflict of interest.

## Footnotes

^1^http://DSPA.predictive.space

^2^http://midas.umich.edu/certificate/approved-courses

^3^https://medicine.umich.edu/medschool/education/phd-programs/about-pibs/pibs-curriculum

## Abbreviations

AdaBoost: Adaptive boosting (ensemble machine learning strategy)
AD: Alzheimer's Disease
ADNI: Alzheimer's Disease Neuroimaging Initiative
API: Application Programming Interface
BANDIT: Bayesian ANalysis to determine Drug Interaction Targets
BDDS: Big Data Discovery Science
CDC: Centers for Disease Control and Prevention
CNN: Convolutional Neural Networks
CSCD: Center for Complexity and Self-management of Chronic Disease
DB: Database
DSPA: Data Science and Predictive Analytics
EDA: Exploratory Data Analysis
GB: Gigabyte
GFT: Google Flue Trends
HCP: Human Connectome Project
HTML5: Hypertext Markup Language (version 5)
JSON: JavaScript Object Notation
MCI: Mild Cognitive Impairment
MIDAS: Michigan Institute for Data Science
MOOC: Massive Open Online Course
NIH: National Institutes of Health
NoSQL: non-SQL (database)
NSF: National Science Foundation
OLAP: Online Analytical Processing
PHP: Hypertext Preprocessor
PIBS: Program in Biomedical Sciences
Q-Q: Quantile-Quantile (plot)
R&D: Research and Development
SDSS: Sloan Digital Sky Survey
SOAP: Simple Object Access Protocol
SOCR: Statistics Online Computational Resource
SQL: Structured Query Language
STEM, Science, Technology: Engineering and Mathematics
SVM: Support Vector Machines
UKBB: United Kingdom Biobank
XML: Extensible Markup Language.

## References

[1] Kreps GL Maibach EW Transdisciplinary science: the nexus between communication and public health. J Commun. (2008) 58:732–48. 10.1111/j.1460-2466.2008.00411.x

[2] Boyack KW Klavans R Börner K Mapping the backbone of science. Scientometrics. (2005) 64:351–74. 10.1007/s11192-005-0255-6

[3] Pfau M Epistemological and disciplinary intersections J Commun. (2008). 58:597–602. 10.1111/j.1460-2466.2008.00414.x

[4] Korazim-Kőrösy Y Mizrahi T Bayne-Smith M García ML Professional determinants in community collaborations: interdisciplinary comparative perspectives on roles and experiences among six disciplines. J Commun Pract. (2014) 22:229–55. 10.1080/10705422.2014.901267

[5] Albert M Paradis E Kuper A. Interdisciplinary promises versus practices in medicine: the decoupled experiences of social sciences and humanities scholars. Soc Sci Med. (2015) 126:17–25. 10.1016/j.socscimed.2014.12.004 25500163

[6] Davies R Trowsdale J The value of instability: lessons from reviewing how and why creativity and the arts might interact with STEM education. Eur J Curric Stud. (2017) 4:584–600. Available online at: http://clok.uclan.ac.uk/21342

[7] Leek JT Jager LR Is most published research really false? Annu Rev Stat Appl. (2017) 4:109–22. 10.1101/050575

[8] Dinov ID. Quant data science meets dexterous artistry. Int J Data Sci Anal. (2019) 7:81–6. 10.1007/s41060-018-0138-6 30923735 PMC6433171

[9] Dinov ID. Volume and value of big healthcare data. J Med Stat Inform. (2016) 4:1–7. 10.7243/2053-7662-4-3 26998309 PMC4795481

[10] Dinov I Heavner B Tang M Glusman G Chard K Darcy M Madduri R Predictive big data analytics: a study of parkinson's disease using large, complex, heterogeneous, incongruent, multi-source and incomplete observations. PLoS ONE. (2016) 1:e0157077 10.1371/journal.pone.0157077 PMC4975403 27494614

[11] Amirian P van Loggerenberg F Lang T editors. *Data Science and Analytics*. In: Big Data in Healthcare. Cham: Springer (2017). p. 15–37. 10.1007/978-3-319-62990-2_2

[12] Dinov I Data Science and Predictive Analytics: Biomedical and Health Applications using R. Computer Science. Cham: Springer International Publishing (2018). 800 p. 10.1007/978-3-319-72347-1

[13] Henly SJ McCarthy DO Wyman JF Heitkemper MM Redeker NS Titler MG. Emerging areas of science: recommendations for nursing science education from the council for the advancement of nursing science idea festival. Nurs Outlook. (2015) 63:398–407. 10.1016/j.outlook.2015.04.007 26187079

[14] Held ML Mallory KC Cummings S Preparing social work students for integrated health care: results from a national study. J Soc Work Educ. (2017) 53:435–48. 10.1080/10437797.2016.1269707

[15] Bangasser DA Rozensky RH Fowler GA Kraha A Lopez AA O'Connor M Psychology's core knowledge, scientific subfields, and health service specialization: preparing a competent workforce-recommendations from the Opening Doors Summit. Train Educ Prof Psychol. (2016) 10:84 10.1037/tep0000117

[16] Fuhrmann CN Halme DG O'Sullivan PS Lindstaedt B. Improving graduate education to support a branching career pipeline: recommendations based on a survey of doctoral students in the basic biomedical sciences. CBE-Life Sci Educ. (2011) 10:239–49. 10.1187/cbe.11-02-0013 21885820 PMC3164563

[17] Pittayachawan S Macauley P Evans T Revealing future research capacity from an analysis of a national database of discipline-coded Australian PhD thesis records. J High Educ Policy Manage. (2016) 38:562–75. 10.1080/1360080X.2016.1196936

[18] van Schalkwyk SC Murdoch-Eaton D Tekian A van der Vleuten C Cilliers F. The supervisor's toolkit: a framework for doctoral supervision in health professions education: AMEE Guide No. 104. Med Teach. (2016) 38:429–42. 10.3109/0142159X.2016.1142517 26998657

[19] Kim MJ Park CG McKenna H Ketefian S Park SH Klopperet H. Quality of nursing doctoral education in seven countries: survey of faculty and students/graduates. J Adv Nurs. (2015) 71:1098–109. 10.1111/jan.12606 25627175

[20] Rahbar MH Dickerson AS Ahn C Carter RE Hessabi M Lindsell CJ. Characteristics of biostatistics, epidemiology, and research design programs in institutions with clinical and translational science awards. Acad Med. (2017) 92:229–36. 10.1097/ACM.0000000000001350 27580435 PMC5263220

[21] Sarkar IN. Biomedical informatics and translational medicine. J Transl Med. (2010) 8:22. 10.1186/1479-5876-8-22 20187952 PMC2837642

[22] Sinche MV Next Gen PhD. Cambridge: Harvard University Press (2016). 10.4159/9780674974791

[23] Kienholz ML Crowleyet RS Bergal JM Levine AS Transformative changes to embrace, manage, and exploit “Big Data” in: Wartman SA, editor. The Transformation of Academic Health Centers. Boston, MA: Elsevier (2015). p. 159–68. 10.1016/B978-0-12-800762-4.00016-5

[24] Treinish LA Scientific Data Models for Large-Scale Applications. New York, NY: IBM TJ Watson Research Center (2004).

[25] Poornima S Pushpalatha M A journey from big data towards prescriptive analytics. ARPN J Eng Appl Sci. (2006) 11. Available online at: http://www.arpnjournals.org/jeas/research_papers/rp_2016/jeas_1016_5099.pdf

[26] Nunes MB Understanding big data for industrial innovation and design: the missing information systems perspective. J Data Inform Sci. (2009) 2:1–6. 10.1515/jdis-2017-0017

[27] Dinov I. Methodological challenges and analytic opportunities for modeling and interpreting big healthcare data. GigaScience. (2016) 5:1–15. 10.1186/s13742-016-0117-6 26918190 PMC4766610

[28] Zhou Y Zhao L Zhou N Zhao Y Marino S Wang T. Predictive big data analytics using the UK biobank data. Sci Rep. (2019) 9:6012. 10.1038/s41598-019-41634-y 30979917 PMC6461626

[29] Sudlow C Gallacher J Allen N Beral V Burton P Danesh J. UK biobank: an open access resource for identifying the causes of a wide range of complex diseases of middle and old age. PLoS Med. (2015) 12:e1001779. 10.1371/journal.pmed.1001779 25826379 PMC4380465

[30] Biobank U UK Biobank: Protocol For A Large-Scale Prospective Epidemiological Resource. Cheshire: UK Biobank Coordinating Centre (2007).

[31] Sporns O. The human connectome: origins and challenges. Neuroimage. (2013) 80:53–61. 10.1016/j.neuroimage.2013.03.023 23528922

[32] Van Essen DC Ugurbil K Auerbach E Barch D Behrens TE Bucholz R. The human connectome project: a data acquisition perspective. Neuroimage. (2012) 62:2222–31. 10.1016/j.neuroimage.2012.02.018 22366334 PMC3606888

[33] Jack CR Bernstein MA Fox NC Thompson P Alexander G Harvey D. The Alzheimer's disease neuroimaging initiative (ADNI): MRI methods. J Magn Reson Imaging. (2008) 27:685–91. 10.1002/jmri.21049 18302232 PMC2544629

[34] Moon S Dinov ID Kim J Zamanyan A Hobel S Thompson PM. Structural neuroimaging genetics interactions in Alzheimer's disease. J Alzheimers Dis. (2015). 48:1051–63. 10.3233/JAD-150335 26444770 PMC4730943

[35] Mason KE Pearce N Cummins S. Associations between fast food and physical activity environments and adiposity in mid-life: cross-sectional, observational evidence from UK Biobank. Lancet Public Health. (2018) 3:e24–33. 10.1016/S2468-2667(17)30212-8 29307385 PMC5764749

[36] Van Essen DC Smith SM Barch DM Behrens TE Yacoub E Ugurbil K. The WU-minn human connectome project: an overview. NeuroImage. (2013) 80:62–79. 10.1016/j.neuroimage.2013.05.041 23684880 PMC3724347

[37] Smith SM Nichols TE. Statistical challenges in “big data” human neuroimaging. Neuron. (2018) 97:263–8. 10.1016/j.neuron.2017.12.018 29346749

[38] Cook S Conrad C Fowlkes AL Mohebbi MH. Assessing google flu trends performance in the United States during the 2009 influenza virus A (H1N1) Pandemic. PLOS ONE. (2011) 6:e23610. 10.1371/journal.pone.0023610 21886802 PMC3158788

[39] Lazer D Kennedy R King G Vespignani A. The parable of Google Flu: traps in big data analysis. Science. (2014) 343:1203–5. 10.1126/science.1248506 24626916

[40] Topol EJ. High-performance medicine: the convergence of human and artificial intelligence. Nat Med. (2019) 25:44–56. 10.1038/s41591-018-0300-7 30617339

[41] Madhukar NS Khade PK Huang L Gayvert K Galletti G Stogniew M A new big-data paradigm for target identification and drug discovery. bioRxiv. (2017) 134973 10.1101/134973

[42] Katsis Y Balac N Chapman D Kapoor M Block J Griswoldet WG Big data techniques for public health: a case study. In: 2017 IEEE/ACM International Conference on Connected Health: Applications, Systems and Engineering Technologies (CHASE). Washington, DC (2017).

[43] Kruskal JB Berkowitz S Geis JR Kim W Nagy P Dreyeret K. Big data and machine learning-strategies for driving this bus: a summary of the 2016 intersociety summer conference. J Am Coll Radiol. (2017) 14:811–7. 10.1016/j.jacr.2017.02.019 28372961

[44] Chang V. Computational intelligence for medical imaging simulations. J Med Syst. (2018). 42:10. 10.1007/s10916-017-0861-x 29177790

[45] Farahani B Firouzi F Chang V Badaroglu M Constant N Mankodiyaet K Towards fog-driven IoT eHealth: promises and challenges of IoT in medicine and healthcare. Future Gener Comput Syst. (2018) 78:659–76. 10.1016/j.future.2017.04.036

[46] Rudy SH Brunton SL Proctor JL Kutzet JN. Data-driven discovery of partial differential equations. Sci Adv. (2017) 3:e1602614. 10.1126/sciadv.1602614 28508044 PMC5406137

[47] Kahn MG Callahan TJ Barnard J Bauck AE Brown J Davidson BN. A harmonized data quality assessment terminology and framework for the secondary use of electronic health record data. Egems. (2016) 4:1244. 10.13063/2327-9214.1244 27713905 PMC5051581

[48] Sahoo SS Nguyen V Bodenreider O Parikh P Minning T Sheth AP. A unified framework for managing provenance information in translational research. BMC Bioinformatics. (2011) 12:1. 10.1186/1471-2105-12-461 22126369 PMC3298549

[49] Dinov I Lozev K Petrosyan P Liu Z Eggert P Pierce J. Neuroimaging study designs, computational analyses and data provenance using the LONI pipeline. PLoS ONE. (2010) 5:e13070. 10.1371/journal.pone.0013070 20927408 PMC2946935

[50] Tonekaboni S Joshi S McCradden MD Goldenberg A What clinicians want: contextualizing explainable machine learning for clinical end use. arXiv:1905.05134 (2019). Available online at" https://arxiv.org/abs/1905.05134

[51] Walliman N Research Methods: the Basics. Oxon: Routledge (2017).

[52] Abouelmehdi K Beni-Hessane A Khaloufi H Big healthcare data: preserving security and privacy. J Big Data. (2018) 5:1 10.1186/s40537-017-0110-7

[53] Atasoy H Greenwood BN McCullough JS. The digitization of patient care: a review of the effects of electronic health records on health care quality and utilization. Annu Rev Public Health. (2019) 40:487–500. 10.1146/annurev-publhealth-040218-044206 30566385

[54] Theobald S Brandes N Gyapong M El-Saharty S Proctor E Diaz T. Implementation research: new imperatives and opportunities in global health. Lancet. (2018) 392:2214–28. 10.1016/S0140-6736(18)32205-0 30314860

[55] Rotman D The Relentless Pace of Automation. MIT Technology Review (2017). Retreived from https://www.technologyreview.com/s/603465/the-relentless-pace-ofautomation (accessed February 2, 2020).

[56] Zakaria F The rise of the rest. Newsweek. (2008) 12:24–31. Available online at: https://www.jstor.org/stable/20032649

[57] Franzoni C Sauermann H Crowd science: the organization of scientific research in open collaborative projects. Res Policy. (2014) 43:1–20. 10.1016/j.respol.2013.07.005

[58] Lange M Stanford Encyclopedia of Philosophy: Progress. (2011). Available online at: http://plato.stanford.edu/archives/spr2011/entries/progress (accessed February 2, 2020).

[59] Blackmar FW The Story of Human Progress, Blackmar FW editor. Leavenworth, WA: Press of Ketcheson & Reeves (1896).

[60] Brue SL Retrospectives: the law of diminishing returns. J Econ Perspect. (1993) 7:185–92. 10.1257/jep.7.3.185

[61] Shephard RW Färe R The law of diminishing returns. Zeitschrift für Nationalökonomie. (1974) 34:69–90. 10.1007/BF01289147

[62] Murphy JE Liles AM Bingham AL Chamberlin KW Dang DK Hill LG Interprofessional education: principles and application. An update from the american college of clinical pharmacy. J Am Coll Clin Pharm. (2019) 1:e17–e28. 10.1002/jac5.1025

[63] Black EW Blue AV Davidson R McCormack WT Using team-based learning in a large interprofessional health science education experience. J Inter Prof Educ Pract. (2016) 5:19–22. 10.1016/j.xjep.2016.09.002

[64] Dinov I Flipping the grant application review process. Stud High Educ. (2019) 1:1–9. 10.1080/03075079.2019.1628201 PMC9528733 36196072

[65] Beddoes S Doctor you: how data will transform health care. Economist. (2018) 426:9–10. Available online at: https://www.economist.com/news/leaders/21736138-welcome-doctor-you-revolution-health-care-coming

[66] Franklin B Advice to a Young Tradesman. Dublin (1820).

